# Supplementary material for: Yeast transformation efficiency is enhanced by TORC1‐ and eisosome‐dependent signaling
Source: Microbiologyopen. 2018 Oct 11;8(5):e00730. doi: 10.1002/mbo3.730 (PMC6528558; doi:10.1002/mbo3.730)
Supplement: Supplementary file 1 [file MBO3-8-e00730-s001.docx]

**Supplementary Table 1:** Mutagenesis primers sequence

|  | **Forward** | **Reverse** |
| --- | --- | --- |
| **Tco89** | ATGGTTCATCGAGGAAGGACTTTGAAGTCAGACACTGATGTAACATCTCTTAAGAAACCATTATTATCATGACA | TCACCTTTGTTGTGGCTGTGTATGCATGTAGCCCACATTTTCCATCCTGC CCTGATGCGGTATTTTCTCCTTAC |
| **Art1** | ATGGCATTTTCACGTCTTACATCTACTCATCAGTCCAATCATAACGGCTATAAGAAACCATTATTATCATGACA | CTACTGGGTTATTCTATTGGAATCTAGAAAATCGGAAAAGTTTTGTATTC CCTGATGCGGTATTTTCTCCTTAC |
| **Npr1** | ATGTCTTCATTAACTCGATTGCTACAGGAAAAACGAAAAAATGAAACTTCTAAGAAACCATTATTATCATGACA | TTATTGATTATTTTGCTTTTTCTTTTTCTTTTCTAGGCCTGCAATATGTGCCTGATGCGGTATTTTCTCCTTAC |
| **Bul1** | ATGGCCAAAGATTTGAACGATTCGGGGTTTCCACCGAAGAGGAAGCCTTTTAAGAAACCATTATTATCATGACA | TTATTTTGTCACTTGCCTAACAGAAATAGGGATATCAATCTTCGCTACGCCCTGATGCGGTATTTTCTCCTTAC |
| **Art3** | ATGCCCATGGACCAATCTATCTCATCTCCATTGTTTCCCATGGAAAAGGATAAGAAACCATTATTATCATGACA | CTAAAGGGTACTCTCATTTATACTTTGTAATCCAGATTCATTATCTAACGCCTGATGCGGTATTTTCTCCTTAC |
| **Seg1** | ATGTTTAGAAGAAGAACAACTGCACCAGAAATGGAACAGGCGGACCCGACTAAGAAACCATTATTATCATGACA | CTATTTCTTTCTACCAAAGATTTTTTTCAGTTTTTTGCCGAAACTACCCTCCTGATGCGGTATTTTCTCCTTAC |
| **Ypk1** | ATGTATTCTTGGAAGTCAAAGTTTAAGTTTGGAAAATCTAAAGAAGAAAATAAGAAACCATTATTATCATGACA | CTATCTAATGCTTCTACCTTGCACCATTGAGCTACCTAGCTGTTCATTTCCCTGATGCGGTATTTTCTCCTTAC |

**Supplementary Table 2:** Diagnostic primers sequence

|  | **Forward** | **Reverse** |
| --- | --- | --- |
| **Tco89** Inside/Outside | GGACGACCTGACTAGAGA | ACCATTTGTCTCCACACC |
| **Tco89** Outside/Outside | TAACCGCAAGGATAGCTAGTTGCG | GAGATACGGAATCCAGCGAAATCG |
| **Art1** Inside/Outside | AACCGTTGATGCTGATGAGGAG | TATGGTGCACTCTCAGTACAATCTGC |
| **Art1** Outside/Outside | TTCGGAGGAGAACGCTGTTG | ACGTGGCAGATTGTTGAAGATATACG |
| **Npr1** Inside/Outside | TATGGGCCGCCTTTAGTTGTATAGAG | AGTTCCAATCCAAAAGTTCACCTGTC |
| **Npr1** Outside/Outside | TATGGGCCGCCTTTAGTTGTATAGAG | GCACTTTCTAAAGCTTCTTTGCTGTG |
| **Bul1** Inside/Outside | GTTCGTGTGTGTCAACAGGTATATCG | TAGATCGGCAAGTGCACAAACAATAC |
| **Art3** Inside/Outside | ACTCCCTTGCGCCATCTATC | GCATCCGCTTACAGACAAGC |
| **Seg1** Inside/Outside | TTTCAGTGCTCCCTTACTTTACCG | GCTTACATCAACACCAATAACGCC |
| **Ypk1** Inside/Outside | ATTACGAACATATCGAATGCGAGCAG | TAATAACAGACATACTCCAAGCTGCC |
